# Editorial: Genetic Studies on Spondyloarthritis: From Disease Predictors to Therapeutic Targets

**DOI:** 10.3389/fgene.2022.859005

**Published:** 2022-02-18

**Authors:** Matteo Vecellio, Fabiana Paladini, Roberta Ramonda

**Affiliations:** ^1^ Nuffield Department of Orthopaedics, Rheumatology and Musculoskeletal Sciences, Botnar Research Centre, University of Oxford, Oxford, United Kingdom; ^2^ Department of Biology and Biotechnology “Charles Darwin”, Sapienza University of Rome, Rome, Italy; ^3^ Unit of Rheumatology, Department of Medicine, DIMED, University of Padua, Padua, Italy

**Keywords:** spondyloarthropathy, genetic, single nucleotide polymorphisms, ankylosing spondylitis, family based association, polygenic risk score, gender

Seronegative spondyloarthropathies (SpA) are a group of chronic inflammatory diseases and ankylosing spondylitis (AS) is the prototype (Linden et al., 1984). In the last decade the clinical diagnosis of axial SpA (axSpA) has been changed, mainly, in the early disease stage for the heterogenous manifestations of the diseases (Ortolan et al., 2021). The Assessment of Spondyloarthritis International Society (ASAS) classification criteria consider axSpA into radiographic (r-axSpA) and non-radiographic (nr-axSpA) (Rudwaleit et al., 2009). SpA main clinical manifestation is the inflammation of the spine and the entheses, but also extra-skeletal manifestations are common, including the inflammation of peripheral joints, the eye (uveitis), the skin (psoriasis) and the gut (inflammatory bowel diseases, IBD) (Elewaut and Matucci-Cerinic, 2009; Bridgewood et al., 2020).

AS is highly heritable (s ∼ 50), with a complex genetic background only partially clarified, undoubtedly dominated by the Human Leukocyte Antigen (HLA-B27) allele, but with >100 genetic loci significantly incriminated by genome-wide association studies (GWAS) (The Wellcome Trust Case Control Consortium, 2007; Ellinghaus et al., 2016). It is now evident that the AS genetic background is shared between the different SpA subfamilies (i.e., psoriatic arthritis, IBD and acute anterior uveitis) contributing to their excess incidence not only in individuals with AS but also in their relatives (Ellinghaus et al., 2016; Robinson et al., 2016; Costantino et al., 2018).

This Research Topic will provide an insight on the current advances in the genetics of SpA, highlighting the candidate genes, pathways and cell types involved in SpA pathogenesis that can be taken forward for designing novel therapeutic approaches and stimulating researchers to investigate the role of genes in the treatment response (Costantino et al., 2018). Furthermore, the impact of ethnicity, genetic role, gender, and environment will be discussed.

Genetics studies are not only those with large datasets (i.e., GWAS). Costantino et al. highlighted the importance and the success obtained from SpA family-based studies, as they have the potential in identifying the different genetic factors involved in SpA. Interest in SpA family-based approaches is renowned as they might be crucial for the identification of rare variants through next-generation sequencing.

Recent GWAS highlighted the different results obtained following the ethnic differences of the cohorts analysed, between Europe and East Asia. Wu et al. illustrated the different genetic distribution (in particular for HLA alleles and IL23R polymorphisms) for Europe and Asia. In addition, the authors emphasized the importance to use a polygenic risk score approach (PRS). Potentially, with PRS it will be possible to have an early diagnose of AS, as PRS will use genotype data from thousands of genetic variants and quantify an individual’s genetic risk. Besides ethnicity, also gender might have an impact on the genetics and epigenetics of SpA susceptibility. Chimenti et al. comprehensively described the genetic associations, the cytokines, the epigenetic mechanisms and the clinical phenotypes which have sex related differences in SpA subfamilies. Ortolan et al. provided a very elegant systematic literature review confirming how genetics might have a major role in predicting response to therapy in SpA, after the analysis of cross-sectional, case-control and cohort studies.

There is an urgent need in the definition of more specific targets for therapy and personalized medicine in SpA. This unmet need is well described by two well-structured articles in our topic. Hromadova et al. discuss the potential in targeting Tyrosine Kinase 2 (TYK2) gene in SpA, with selective TYK2 inhibitors. The authors covered the different aspects of TYK2 from basic biology to therapeutic targeting, especially in SpA. The current available treatment in axial psoriatic arthritis (axPsA) is the focus of the work by Floris et al. The authors summarized the recent findings in axPsA focusing on the efficacy of the currently available treatments including TNFα, IL17/IL23 and JAK inhibitors.

Simone et al. explored the dysregulated “type17” response in SpA. The authors elegantly summarised the multifaceted role of Th17 cells as possible trigger occurring in SpA, covering the genetics, environment and microbiome aspects of these cells.

The specific functional role of SpA-associated Single Nucleotide Polymorphisms (SNPs) has been analysed in two works from our collection. Pimenta et al. analysed four SNPs in Alpha-actinin-3 (ACTN3) and Vitamin D receptor (VDR) genes to demonstrate association between axSpA with muscle performance, analysing clinical and epidemiological data. Finally, Cohen et al. used a functional genomics approach, including *in silico* analysis, electrophoretic mobility shift assay and chromosome conformation capture, to define the functional role of a strongly AS-associated SNP located at the genomic locus encompassing *RUNX3*, a transcription factor involved in the development of CD8^+^ lymphocytes.

In conclusion, we can firmly affirm that all the articles included in this collection provided a clear evidence of the importance of genetics studies in SpA and the need of genetics in the definition of new targets for therapy and improve drug development.

## References

[B1] Bridgewood C. Sharif K. Sherlock J. Watad A. McGonagle D. (2020). Interleukin-23 Pathway at the Enthesis: The Emerging story of Enthesitis in Spondyloarthropathy. Immunol. Rev. 294 (1), 27–47. 10.1111/imr.12840 31957051

[B2] Costantino F. Breban M. GarchonGenetics H.-J. Genomics F. (2018). Genetics and Functional Genomics of Spondyloarthritis. Front. Immunol. 9, 2933. 10.3389/fimmu.2018.02933 30619293 PMC6305624

[B3] Elewaut D. Matucci-Cerinic M. (2009). Treatment of Ankylosing Spondylitis and Extra-articular Manifestations in Everyday Rheumatology Practice. Rheumatology 48 (9), 1029–1035. 10.1093/rheumatology/kep146 19561158

[B4] Ellinghaus D. Jostins L. Jostins L. Spain S. L. Cortes A. Bethune J. (2016). Analysis of Five Chronic Inflammatory Diseases Identifies 27 New Associations and Highlights Disease-specific Patterns at Shared Loci. Nat. Genet. 48 (5), 510–518. 10.1038/ng.3528 26974007 PMC4848113

[B5] Linden S. V. D. Valkenburg H. A. Cats A. (1984). Evaluation of Diagnostic Criteria for Ankylosing Spondylitis. Arthritis Rheum. 27, 361–368. 10.1002/art.1780270401 6231933

[B6] Ortolan A. Kiltz U. Doria A. Aggarwal A. Ramonda R. (2021). Do we Believe in Non-radiographic Axial Spondyloarthritis? A Debate. Autoimmun. Rev. 20 (1), 102703. 10.1016/j.autrev.2020.102703 33188919

[B7] Robinson P. C. Leo P. J. Leo P. J. Pointon J. J. Harris J. Cremin K. (2016). The Genetic Associations of Acute Anterior Uveitis and Their Overlap with the Genetics of Ankylosing Spondylitis. Genes Immun. 17 (1), 46–51. 10.1038/gene.2015.49 26610302

[B8] Rudwaleit M. van der Heijde D. Landewé R. Listing J. Akkoc N. Brandt J. (2009). The Development of Assessment of SpondyloArthritis International Society Classification Criteria for Axial Spondyloarthritis (Part II): Validation and Final Selection. Ann. Rheum. Dis. 68 (6), 777–783. 10.1136/ard.2009.108233 19297344

[B9] The Wellcome Trust Case Control Consortium (2007). Genome-wide Association Study of 14,000 Cases of Seven Common Diseases and 3,000 Shared Controls. Nature 447, 661–678. 10.1038/nature05911 17554300 PMC2719288

